# Extracellular Vesicles in Bone Tumors: How to Seed in the Surroundings Molecular Information for Malignant Transformation and Progression

**DOI:** 10.3389/fonc.2021.722922

**Published:** 2021-09-20

**Authors:** Alfredo Cappariello, Nadia Rucci

**Affiliations:** Department of Biotechnological and Applied Clinical Sciences, University of L’Aquila, L’Aquila, Italy

**Keywords:** bone tumor, extracellular vesicles, vicious cycle, dormancy, drug resistance, premetastatic niche

## Abstract

Bone is a very dynamic tissue hosting different cell types whose functions are regulated by a plethora of membrane-bound and soluble molecules. Intercellular communication was recently demonstrated to be also sustained by the exchange of extracellular vesicles (EVs). These are cell-derived nanosized structures shuttling biologically active molecules, such as nucleic acids and proteins. The bone microenvironment is a preferential site of primary and metastatic tumors, in which cancer cells find a fertile soil to “seed and blossom”. Nowadays, many oncogenic processes are recognized to be sustained by EVs. For example, EVs can directly fuel the vicious cycle in the bone/bone marrow microenvironment. EVs create a favourable environment for tumor growth by affecting osteoblasts, osteoclasts, osteocytes, adipocytes, leukocytes, and endothelial cells. At the same time other crucial tumor-mediated events, such as the premetastatic niche formation, tumor cell dormancy, as well as drug resistance, have been described to be fostered by tumor-derived EVs. In this review, we will discuss the main body of literature describing how the cancer cells use the EVs for their growth into the bone and for educating the bone microenvironment to host metastases.

## Extracellular Vesicles: An Introduction

### Biogenesis of EVs

EVs are nanosized structures actively released by all cells (1). They are complex phospholipidic bi-layers distinguishable in subtypes accordingly to their dimension: small EVs range below 100 nm (also known as exosome or exosome-like vesicles), middle/large EVs size between 100 and 1000 nm (also known as microvesicles and shedding vesicles), and finally apoptotic bodies, with a diameter ranging from 800 to 5000 nm (2). Nowadays apoptotic bodies are considered a stand-alone class of EVs, due to their peculiar biogenesis and biological functions, being mainly involved in programmed cell death. However, recent evidence has recognized an immunomodulatory role for these particles (1–3). Besides the size, the most important difference between the small and medium/large EVs lies in their biogenesis (4). In fact, small EVs arise from the fusion of endocytic vesicles sorted in a multivesicular body (MVB) *via* a complex molecular machinery involving endosomal sorting complex required for transport (ESCRT) components, ceramide/sphingomyelinase pathway members, and Ras-related proteins in brain (RAB) proteins (4). This MVB then fuses with the plasma membrane and releases the small EVs in the extracellular space. Medium/large EVs arise directly from the plasma membrane *via* ADP-ribosylation factor (ARF) 6-mediated activation of phospholipase D (PLD), resulting in the recruitment of extracellular signal-regulated kinase (ERK) and phosphorylation of the myosin light-chain kinase (MLCK). This cascade triggers the budding of the cellular membrane, culminating in the formation and release of EVs from the cell (4). During their biogenesis, EVs entrap different macromolecules, such as nucleic acids (DNA, mRNA, miRNAs, long non-coding RNAs), lipids, and proteins (cytosolic factors, receptors, and ligands), which are then transferred to target cells where they induce metabolic changes (1).

### Paracrine and Systemic Effects of EVs

Once EVs reach a target cell, the physical/molecular interactions between EV and cell membranes activate the EV uptake. This interaction has been shown to occur *via* multiple routes, including a direct fusion between EVs and the plasma membrane (5), as well as EV internalization *via* lipid draft-, clathrin-, and calveolae-dependent endocytosis, macropinocytosis, and phagocytosis (6–8). Indeed, the route of EVs uptake is likely dependent on the following factors: lipid and protein composition of the released EVs and of the plasma membrane of recipient cells, EV subtype, cell metabolic status, and extracellular space conditions (i.e. pH, oxygen tension, and extracellular matrix components).

The contribution of EVs in the paracrine and distant communication is easily conceivable, since EVs can be found in all biological fluids and bloodstream and can reach every site of the body. However, to demonstrate these events is quite challenging. Lai et al. addressed this point by staining EVs with multiplexing bioluminescent (*Gaussia* luciferase, Gluc) and fluorescent (enhanced green fluorescent, EGFP, and tandem tomato, tdTomato) reporters into the mouse thymoma cell line EL4, finding that EV uptake and delivery of the mRNAs to recipient cells occurred within 1 hour (9). Furthermore, seeding EL4 cells in diffusion chambers, which are then subcutaneously implanted in the dorsal skin of mice, confirmed the systemic distribution of released EVs (9). Similar evidence was reported in another study, where EVs visualization and EVs cargo transfer between tumor cells and stromal cells in mice was assessed by high-resolution intravital imaging. Based on the Cre-LoxP system, the physiological effects of this exchange were studied in different melanoma and mammary tumor models (10). By generating Cre-expressing B16 melanoma cells and injecting them in mice expressing the Cre-LoxP reporter tdTomato, EVs release and uptake to distant organs (i.e., lymph nodes, lungs, and spleen) were observed within 2 weeks. In the same work, the authors showed that EVs released by eGFP^+^ MDA-MB-231 breast cancer cells orthotopically injected in mice were taken up by the less malignant T47D tumor cells located into the contralateral mammary pad. Moreover, these EVs carry mRNAs involved in migration and metastasis. In fact, T74D cells that incorporated MDA-MB-231-EVs metastasized to lung 7.9-fold more than control cells (10). Later on, You and colleagues exploited the intrinsic optical properties of tissues and tumor-derived EVs based on their NADH content (11). In fact, they reached a label-free visualization and characterization of metabolic fingerprint of EVs on a multiphoton microscopy *via* 2-photon fluorescence of FAD, 3-photon fluorescence of NAD(P)H, and third harmonic generation for the structural properties (lipid–water interface) of the EVs. With this method, EVs located inside fresh human breast biopsies collected from invasive ductal carcinoma patients were analyzed and compared to EVs collected from biopsies of healthy women subjected to breast reduction surgery, finding that the former presented with a higher NAD(P)H amount. Moreover, the concentration of NAD(P)H-rich EVs allowed not only to discriminate between patients and healthy subjects, but also to stratify the former according to tumor staging. However, this approach failed to reveal the dynamics of EVs in the tumor microenvironment, showing EVs firmly attached to the extracellular matrix and vessel walls, both in human biopsies and in animal models of breast tumor (11).

More recently, de Jong et al. described a CRISPR-Cas9-based reporter system to *in vitro* trace the EV-shuttling of small non-coding RNA molecules at single-cell resolution (12). They applied this technique on different breast cancer cell lines and demonstrated the functional transfer of small RNAs by means of EVs within 5 days. Moreover, knocking down genes involved in EV biogenesis and trafficking, such as Alix, Rab27a, Pak1, Rac1, Cav1, and RhoA, decreased EV uptake, while the silencing of Ankyrin Repeat And FYVE Domain Containing (ANKFY1), involved in vesicle transport, increased EV uptake (12).

## Extracellular Vesicles in Bone Physiology

Bone is an active tissue in which different cell types live together and their crosstalk guarantees the mechanical, biochemical, and hormonal functions of the skeleton (13, 14). A fine cell-to-cell communication exists by means of membrane-bound and soluble molecules, allowing a selective spatial cellular differentiation and activity (15). The pivotal functional bone unit is the “bone remodeling unit” (BRU), constituted by bone forming osteoblasts and bone resorbing osteoclasts (16). Their activity is tightly coordinated by two main cytokines expressed by osteoblasts: macrophage-colony stimulating factor (M-CSF) and receptor activator of NF-κB Ligand (RANKL), having their receptor on pre-osteoclasts/monocytes: the colony stimulating factor 1 receptor (CSF1R, *alias* c-fms) and RANK, respectively (17). Once mature osteoclasts have resorbed the bone, a plethora of molecules are released from the extracellular matrix, affecting the osteoblast function and differentiation, such as transforming growth factor (TGF)-β, connective tissue growth factor (CTGF), osteocalcin (OCN), osteopontin (OPN), bone morphogenetic proteins (BMPs), insulin-like growth factor (IGF)-1 and -2, platelet-derived growth factor (PDGF), and calcium ions (18, 19). Beside these molecules, many others concur to regulate this cellular crosstalk, such as stromal derived factor (SDF)-1, interleukin (IL)-1 and -6, fibroblast growth factor (FGF), parathyroid and parathyroid hormone related peptide (PTH/PTHrP), Lipocalin-2 (LCN2), NOTCH family members, Ephrin ligands and receptors, Semaphorins, and vascular endothelial growth factors (VEGFs), that establish the so called “virtuous cycle of the bone” (20–23). In addition, the BRU establishes a further crosstalk with the bone/bone marrow-resident cells thorough juxtacrine and paracrine communications (24–26). Indeed, EV involvement in bone physiology has been elucidated in many aspects. The very first observation for a contribution of EVs in the process of bone mineralization was reported by two different groups in late ‘60s (27, 28). Later on, EVs from osteoblasts were demonstrated to bind, by means of annexin 2, calcium phosphate, and other ions, thus triggering the formation of nuclei of mineralization in the bone matrix (29). Moreover, EVs have been described to be active coupling factors in osteoblast-osteoclasts crosstalk. In fact, it is known that osteoblasts secrete EVs shuttling RANKL, which in turn sustains *in vitro* osteoclast formation (30). In line with this data, we found that osteoblast-derived RANKL-positive EVs injected in osteoclast-poor RANKL knock out mice induced the osteoclastic commitment and the appearance of tartrate-resistant acid phosphatase (TRAP) positive cells in these mice (31). These results demonstrate another way exploited by osteoblasts to regulate osteoclast formation besides the juxtacrine and paracrine signaling. *Vice versa*, osteoblast differentiation and function can be also regulated by osteoclasts, as shown by Ma et al., who found that apoptotic bodies released by mature resorbing osteoclasts trigger osteoblast differentiation by activating the RANKL reverse signaling in these cells (32, 33). The pro-osteoblast differentiating effect could be also accomplished by an autocrine mechanism, since it has been demonstrated that EVs from mineralizing osteoblasts promote their own differentiation by activating Wnt- β-catenin pathway, while reducing Axin1 expression in bone marrow stromal cells (34). Recent reports show that osteocytes, the most abundant cells in bone having not only a mechanosensing role but also a regulatory role on osteoclast and osteoblast differentiation, accomplish these functions by releasing EVs. As a matter of fact, Morrel et al. found that under mechanical stress osteocytes release EVs, which in turn enhance *in vivo* bone formation (35). At the same time, osteocyte homeostasis is regulated by EVs, as demonstrated by Ren et al., who found that exosomes from adipose tissue MSCs counteracted the hypoxia- and serum deprivation-induced apoptosis of the osteocyte-like cell line MLO-Y4, eventually leading to a lower production of RANKL by these cells and, consequently, to an inhibition of osteoclast formation (36). Consistently, Lu and colleagues found that exosomes isolated from adipose tissue-derived MSCs promoted *in vitro* proliferation and osteogenic differentiation of primary human osteoblasts. Moreover, pre-conditioning of the donor cells with tumor necrosis factor (TNF)-α increased the pro-osteoblastogenic effect by a Wnt signaling dependent mechanism (37). Finally, endothelial cells also contribute to bone homeostasis by releasing EVs, which induce osteogenic differentiation of MSCs by shuttling Galectin-3 (38).

## Extracellular Vesicles Contribution to Tumor Colonization of Bone

Neoplastic bone diseases are fueled by EVs (39). As a matter of fact, the onset and progression of both primary and metastatic bone cancers are promoted by a massive release of EVs, characterized by an abnormal molecular cargo (40). This event is not surprising considering that many key mechanisms involved in cancer progression, such as microenvironment acidification or aberrant pathways activation, also induce EVs production (41). In the bone context, tumor cells exploit the molecular pathways involved in bone remodeling to their own advantage, thus converting this “virtuous cycle” in a “vicious” one (42).

Different types of tumors can thrive in the bone *milieu*: primary tumors such as osteosarcoma (OS), Ewing’s sarcoma, chondrosarcoma or fibrosarcoma and, very frequently, metastatic tumors from breast, prostate, lung, renal, colon, and bladder cancers (43, 44). All these tumors can hijack physiological stimuli to their advantage. Key examples are SDF-1, RANKL, and OPN, which can be exploited by cancer cells due to their chemoattractant and pro-mitogenic activities, that summoning and promoting the engraftment of cancer cells into the bone microenvironment (45). The interaction between tumor cells and the bone induces a deregulation of bone giving rise to bone lesions can be classified as osteosclerotic, characterized by osteoblastic overactivation, osteolytic, due to an exacerbated osteoclast function, and mixed, when both features coexist in the same site (46, 47). Indeed, OS can generate all the three types of lesions, due to a wide histologic variety: the majority of OS cases (>85%) has osteosclerotic features, telangiectatic OS (~7%) presents osteolytic lesions, while small cell OS (~2%) produces mixed lesions (48, 49). In contrast, breast and lung cancers usually give rise to osteolytic bone metastases, while osteosclerotic lesions characterize most of prostate cancer-induced bone metastases (47–50).

### Extracellular Vesicles in Bone Tumors Growth

**Figure 1:**
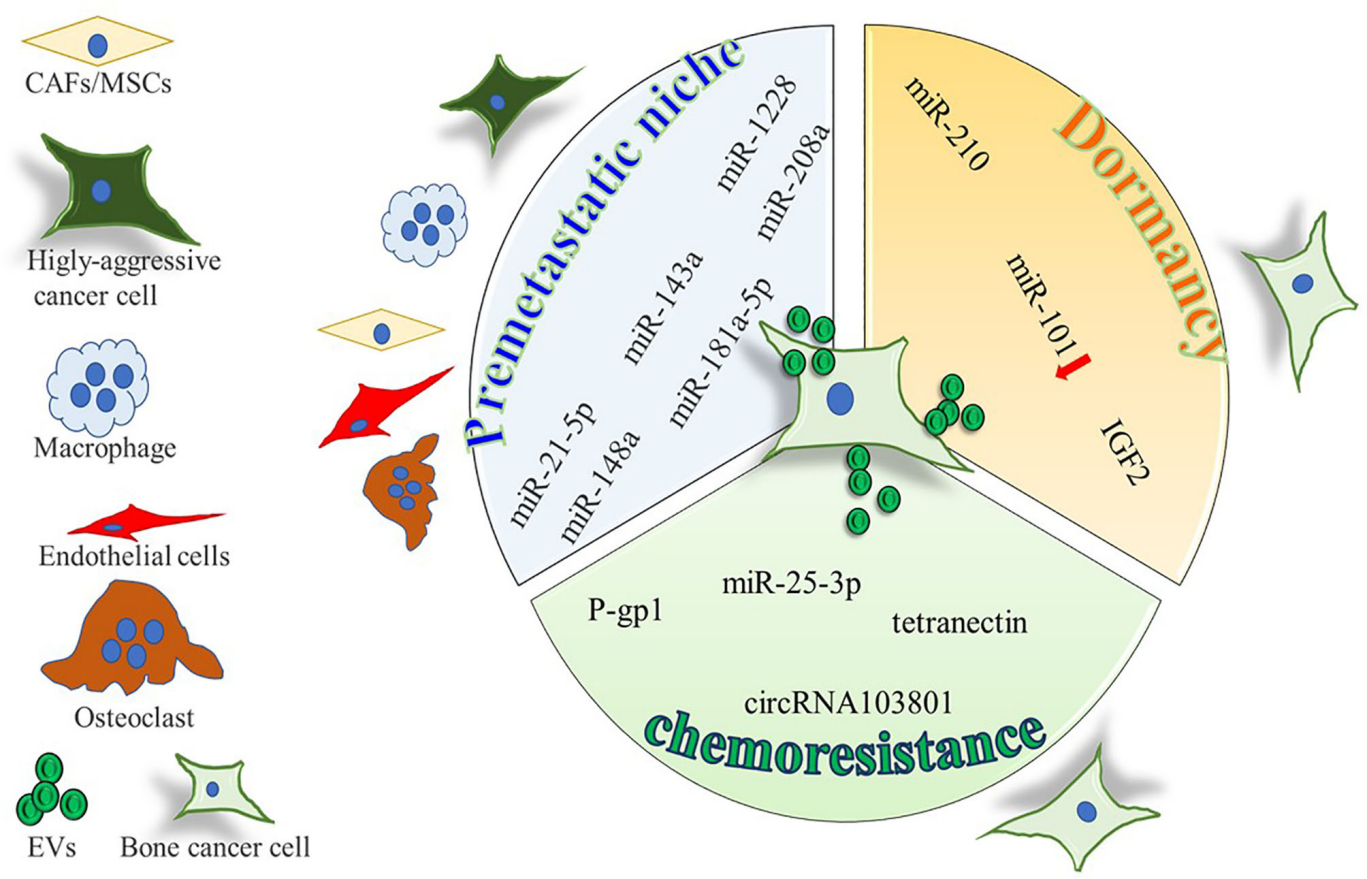
Cartoon summarizing the intercellular molecular cargo exchanged by means of EVs in the context of primary bone tumors, between tumor cells and resident cells. Only molecules shuttled by EVs were indicated, all positively involved in premetastatic niche formation, tumor dormancy, and chemoresistance, except for miR-101, inversely correlated with dormancy potential.

OS is the most common primary tumor of bone, predominantly occurring in adolescents, with a second peak in elderly adults (51). MSCs and osteoblast precursors undergo malignant transformation, eventually leading to the deposition of an aberrant, immature bone (52). As for other types of tumors (**Figure 1** and **Table 1**), growing evidence demonstrates a crucial impact of EVs on OS development. An interesting work by Macklin and colleagues demonstrated that the OS aggressive behavior can be transferred from the highly metastatic KHOS OS cell line to a non-metastatic KHOS subtype by means of EVs (67). This effect was due to an enrichment, inside the EVs, of molecules involved in G-protein coupled receptor signaling. Moreover, the highly metastatic OS cells secrete 3-fold more vesicles than the lower metastatic ones, while multiphoton microscopy with fluorescence lifetime *in vivo* imaging demonstrated a preferential seeding of lungs by EVs derived from the highly metastatic OS clonal variants (67). Interestingly, Qi et al. showed that proliferation of the MG63 OS cell line is supported by EVs derived from bone marrow stromal cells, eventually leading to the activation of the Hedgehog pathway in the recipient cells (68), while other studies found that aggressiveness and metastatic potential of OS are directly related to the release of soluble and EV-bound urokinase plasminogen activator (uPA) as well as EV-bound to PD-L1/N-cadherin (69, 70).

**Table 1:** Extracellular vesicles in the premetastatic niche in bone and in osteotropism provision.

Donor	EV-Mediator	Target	Effect	Refs
Osteosarcoma cells U2-OS, SAOS2, MG-63	miR-148a,miR-21-5p	Osteoclasts,endothelial cells	TRAP and MMP9 upregulation,VEGF, IL-6, -8 upregulation	(53)
SAOS2	miR-21-5p, miR-143-3p,miR-148a-3p and 181a-5p	MG63	LOX, TIMP3 increase	(54)
CAFs	miR-1228	MG-63	SCAI suppression	(55)
MSCs	miR-208a	SAOS2, MG63	PDCD4 suppression	(56)
Osteosarcoma LM7vcells	Whole EVs	Macrophages	Tgfb2, CCL2	(57)
143-B, SAOS2	Whole EVs	Alveolar macrophages	Immune cells recruitment	(58)
Melanoma cells LCP	Whole EVs	Melanoma cells WM-266,SK-Mel28	CXCR7 upregulaiton	(59)
NSCLC, CRL-2868 lung cells	Amphiregulin	osteoclasts	EGFR induction	(60)
Lung A549	miR-192	Endothelial cells	angiogenesis	(61)
Breast cancer cells SCP28	miR-21	Osteoclasts	PDCD4 repression	(62)
Prostate cancer cells C4	miR-940	Osteoblasts	ARHGAP1/FAM134A repression	(63)
Prostate tumor cells TRAMP-C1	Whole EVs	Osteoclast	Osteoclast differentiation decrease	(64)
Prostate cancer cells PC-3	Whole EVs	Osteoclasts	miR-214 and p-p65 downregulation	(65)
Prostate cancer cellsDu145 and PC3	TGF-β	Adipose cells	VEGF-A, HGF, MMPs induction	(66)

OS derived-EVs are also able to influence bone resident cells to promote OS grown. As an example, OS derived EVs stimulate endothelial cells to increase the secretion of IL-6,-8 and VEGF, by shuttling miR-148a and miR-21-5p (53). Osteoclasts are important players in OS, whose number seems to be correlated with tumor aggressiveness and metastatization (71). Interestingly, Garimella et al. found that EVs isolated from the 143B OS cell line contain a pro-osteoclastogenic cargo including matrix metalloproteinases-1 and -13 (MMP-1, -13), TGF-β, CD-9, and RANKL, thus suggesting a role for these EVs in mediating bone degradation (72). Similarly, our recent work shows that EVs isolated from the MNNG/HOS human OS cell line significantly impair osteoblast differentiation while increasing osteoblast secretion of pro-osteoclastogenic/inflammatory cytokines (i.e., IL-6, Lcn2, RankL, CCL2,5,6,12, and CXCL1,2,5) and of MMP3. Moreover, OS-derived EVs have a proangiogenic effect, evaluated both *in vitro* and *in vivo* (73).

Ewing’s sarcoma is the second most common bone cancer in children and adolescents. It presents with small round cells derived from the neural crest and is usually associated with the chimeric fusion gene Ewing sarcoma breakpoint region 1/Friend leukemia integration 1 transcription factor (EWSR1/FLI1) (74). Miller et al. firstly described that Ewing’s sarcoma-released EVs are enriched in the mRNA of the chimeric fusion gene, along with Six transmembrane epithelial antigen of the prostate 1 (STEAP1) and Lipase, member 1 (LIP1) mRNA (75). Interestingly, the specificity of this EV cargo could make circulating EVs suitable biomarker candidates for patients.

Chondrosarcoma accounts for approximately 20% of bone tumors and is due to a malignant transformation of chondroblasts (76). From an histological point of view, it is classified in three stages according to cytonuclear atypia, number of multinucleated cells, degeneration of chondroid matrix, and absence of mitosis. Also for this tumor, a contribution of EVs in its progression has been hypothesized. As an example, the SW1353 chondrosarcoma cell-derived EVs shuttle the long non-coding RNA (lncRNA) RAMP2-AS1, which acts as a molecular decoy for miR-2355-5p to regulate VEGFR2 expression thus increasing the angiogenic ability of HUVEC endothelial cells (77).

Finally, also the aggressiveness of fibrosarcoma seems to be supported by EVs release. This is a very rare bone tumor (0.5 cases in a million per year) caused by malignant transformation of fibroblasts (78). Malignant cells are strongly positive for vimentin and have an altered production of collagen, which is inversely correlated to the histological grade (78). Hakulinen et al. found that EVs isolated from the human fibrosarcoma cells HT‐1080 contain both the inactive cleaved and the active full-length forms of MT1-MMP, which in turn activate pro-MMP2, allowing type I collagen and gelatin degradation (79).

### Extracellular Vesicles in Metastasis Development

OS frequently metastasizes to lungs and its aggressiveness is related to osteoclastogenesis and neo-angiogenesis (80, 81). Likewise, it has been demonstrated that OS derived EVs increase both osteoclasts and neo-angiogenesis, by shuttling miR-148a and miR-21-5p to target cells (53). Jerez et al. investigated whether EV cargo can be predictive for metastatic potential of OS by investigating the miRNAs profile of EVs from metastatic SAOS2 and non-metastatic MG63 osteosarcoma cell lines (82). They found that SAOS2-EVs were particularly enriched in miR-21-5p, miR-143-3p, miR-148a-3p, and 181a-5p compared to MG63-EVs. Consistently, bioinformatic analysis revealed, among the miRNA targets, some genes involved in ECM remodeling, such as the collagen-crosslinking enzyme lysyl oxidase (LOX), and the tissue inhibitor metalloproteinase 3 (TIMP3), suggesting that SAOS2 related miRNAs may influence metastatic potential of OS at least in part by modulating ECM remodeling (82). Interestingly, another study highlights the protumoral role of cancer associated fibroblasts (CAFs) on OS by releasing EVs enriched in miR-1228. Once taken up by the OS cell lines MG-63 and HOS, miR-1228 suppressed *SCAI* (suppressor of cancer cell invasion), resulting in the promotion of migration and invasion of OS cells (83). Of note, MSCs have also been described to support osteosarcoma progression by releasing EVs (84). In particular, MSC-EVs enriched in miR-208a are taken up by SAOS2 and MG63 cells, which inhibited programmed cell death 4 (PDCD4) eventually stimulating tumor cells proliferation, migration and invasion (84).

Once reached the metastatic site, which is usually in the lungs, OS cells regulate the function of resident cells by means of EVs. Indeed, it has been reported that the LM7 metastatic osteosarcoma cell line releases EVs able to induce the production of IL-8, TGF-β2 and CCL22 from alveolar macrophages and the impairment of the macrophagic immune-surveillance function by promoting a switch of the M1-to-M2 phenotype (85). Interestingly, Mazumadar and colleagues found that EVs from the human OS cell lines 143B and SAOS2 (highly metastatic and non-metastatic, respectively) when injected intraperitoneally into SCID mice promote the recruitment of CD11+ Gr+ immune cells into lungs. Moreover, OS-derived EVs alone can recapitulate myeloid cell infiltration in the lungs of naïve mice but are insufficient to promote the development of OS metastasis, thus indicating that the establishment of the PMN in the lungs may require a combination of tumor-secreted factors along with EVs (86).

**Figure 2:**
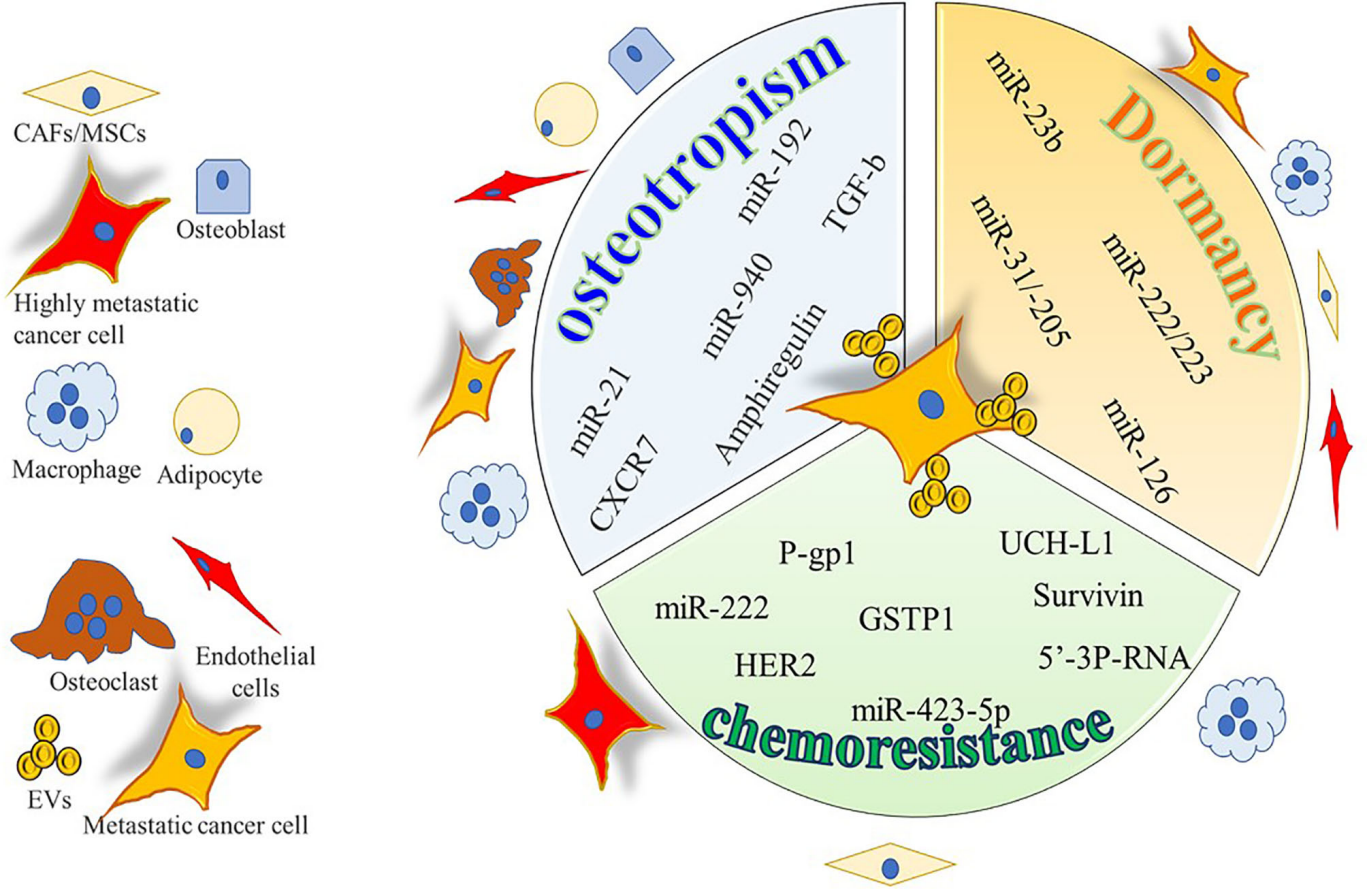
Cartoon summarizing the intercellular molecular cargo exchanged by means of EVs between tumor cells and resident cells in bone metastases. Only molecules encompassed in EVs were indicated and reported to be positively involved in osteotropism, tumor dormancy, and chemoresistance.

As already mentioned, to engraft and colonize a distant organ, cancer cells are able to influence the future host tissue by preparing the resident cells to receive tumor cells and support their growth (87). This process, generating a microenvironment suitable for cancer dissemination before the dissemination itself occurs, is called “premetastatic niche” (88). Many mechanisms and secreted factors inducing the premetastatic niche (PMN) have been discovered (89) and undoubtedly EVs are important players in this process. This is also true for bone metastases which, as already stated, represent the preferential secondary site of growth for several tumors. The accumulation of EVs in a metastatic site is accompanied by several modifications in the microenvironment, such as resident cells activation, matrix deposition and vascular proliferation (**Figure 2** and **Table 1**).

Prostate cancer (PrCa) is the tumor with the highest propensity to metastasize to bone. In PrCa-induced bone metastases, several reports have emerged, showing the crucial role of tumor-derived EVs cargo in the preparation of the “soil”. Hashimoto et al. found that miR-940 is highly expressed in EVs from highly osteotropic prostate cancer cells, and promotes osteoblastic/osteosclerotic bone lesions by targeting Rho GTPase Activating Protein 1 (ARHGAP1) and family with sequence similarity 134 member A (FAM134A) (90). Consistently, Karlsson et al. found that EVs from the TRAMP-C1 prostate tumor cell line impair osteoclast formation, while enhancing abnormal bone formation at the metastatic site (91). On the same line, other authors showed that PC3 PrCa cells decrease osteoclasts formation by means of EVs, which reduce the expression of miR-214 and the phosphorylation of p65 in osteoclast cells, finally suppressing the NF-κB signaling and osteoclast differentiation (92).These events enhance angiogenesis and finally tumor proliferation and invasiveness. PrCa cells also affect bone marrow MSCs behavior by means of EVs, by promoting a shift from an adipogenic toward a myofibroblastic differentiation. The latter cells in turn secrete high levels of pro-angiogenic (VEGF-A) and pro-invasive factors like MMP-1,-3 and -13 (93). Consistently PrCa cell (i.e., C4‐2B, PC‐3 and RWPE‐1)-derived EVs contribute to tumorigenic reprogramming of MSCs by delivering oncogenic transcripts of H‐Ras, K‐Ras and oncomiRNAs miR‐125b, miR‐130b, and miR‐155 as well as the Ras superfamily of GTPases Rab1a, Rab1b, and Rab11a (94). Finally, an autocrine effect of tumor cell-derived EVs was also demonstrated by Dai and Gao, who found that miR-183 shuttled by PC3 derived EVs promotes cancer cell proliferation, migration and invasion through the inhibition of Tropomyosin 1 (TPM1) (95).

With regards to breast cancer (BrCa), it also very frequently metastasizes to bone, inducing osteolytic lesions. EVs released by the SCP28 BrCa cells are enriched in miR-21 which is internalized by the osteoclasts (96). Inside these cells miR-21 represses PDCD4, a negative regulator of osteoclastogenesis, leading to an exacerbation of the osteoclast function. In agreement with these observations, breast cancer patients with bone metastasis showed higher expression of miR-21 in serum exosomes than those without metastasis or with non-bone metastasis (97).

Hypoxic BrCa cells 4T1 release miR-210-containing EVs in the tumor microenvironment resulting in inhibition of Ephrin-A3 and Protein tyrosine phosphatase 1B (PTP1B), as well as in the increase of angiogenesis and tumor proliferation (98). BrCa-associated fibroblasts secrete EVs that activate the Wnt-planar cell polarity (Wnt-PCP) and finally promote dissemination of cancer (99). Finally, we recently demonstrated that MDA-MB-231 derived-EVs affect bone cells *in vitro*, since they increase osteoclast formation while dramatically impairing osteoblast differentiation and activity. Moreover both *in vitro* (tube formation assay) and *in vivo* (Matrigel plug assay) experiments demonstrated the ability of these EVs to promote angiogenesis (100).

Melanoma is a very aggressive tumor, frequently generating visceral metastasis (101) while bone metastases occurr in around 15% of patients, with a dramatic worsening of quality of life and survival (102). An *in vitro* study from Mannavola and colleagues showed that the osteotropic melanoma LCP cells release EVs able to switch the non osteotropic WM-266 and SK-Mel28 cell lines to an osteotropic one, by increasing the expression of CXCR7 (103). In contrast, the expression of α_6_β_4_ or α_v_β_5_ integrins favors a specific affinity for the lung and the liver, respectively (54). In line with these observations, integrins β_4_ and α_v_ expression in EVs isolated from the plasma of melanoma patients predict metastasis to lung and liver (54).

Lung cancer can also metastasize to the bone, and EVs facilitate this event (55). Indeed, EVs from the CRL-2868 non-small cell lung cancer (NSCLC) cell line and from lung cancer patients contain Amphiregulin (AREG) which in turn promotes osteoclast differentiation and exacerbated bone resorption by an EGFR-dependent mechanism (56). In another study, metastatization to bone of lung adenocarcinoma A549 cells is reported to be inversely correlated miR-192 expression (57). In particular, the authors found that EVs from the highly metastatic A549-M1 subclone presented with a lower content of miR-192 compared to A549 parental EVs. Interestingly, intravenous administration of miR-192-enriched EVs the day before the intracardiac injection of the A549-M1 subclone, drastically reduced the bone metastatic burden of these cells in the tibia, as well as the number of CD31 positive cells in comparison to naïve EVs (57).

## Extracellular Vesicles Contribution to Tumor Cell Dormancy in the Bone

Once cancer cells reach a distant organ, they can encounter a dormancy-permissive environment (58). In particular, the resident stem cell niche compartment can favors the survival of a distant cancer stem cell, by the same mechanisms usually employed to regulate the maintenance of the stem cell pool and the recruitment of uncommitted cells into the tissue (59, 104, 105). On the other side, the dormant tumor cells can acquire stem cell-like properties overexpressing specific genes to interact with the niche (46). As an example, BrCa cells can shift toward a dormant phenotype in the endosteal niche, activating a hematopoietic stem cell profile *via* the Notch2 pathway (106). Cancer dormancy accounts for a considerable clinical problem, since it is responsible for tumor recurrence; in fact the tumor awakening leads to the generation of secondary tumor lesions, which in many cases constitute the cause of the death of patients (107).

Several studies revealed that EVs can also trigger the dormant phenotype of tumor cells (**Figure 1** and **Table 2**). Kling et al. found that under hypoxic conditions, the Ewing’s sarcoma cells A673 and SK-ES-1 upregulate and load miR-210 in their EVs, which suppresses Caspase 8 Associated Protein 2 (CASP8AP2) expression in parental cells, eventually inhibiting OS cells apoptosis and inducing a stem-like phenotype (60). Furthermore, OS modulate its aggressiveness and dormant phenotype by the EV-shuttled miR-101 (61). In fact, *in vivo* injection of SAOS2 and SOSP-9607 cells overexpressing miR-101 resulted in less lung metastases in mice, through the silencing of B cell lymphoma protein, BCL6. Consistently, when miR-101 was overexpressed in MSCs, the MSC-EVs were enriched in this miRNA and effectively reduced *in vitro* migration and invasion of SAOS2 and 143B cells. These effects have been confirmed in immunocompromised mice, in which the treatment with MSC-EVs-miR-101 reduced the lung metastatic foci after 2 weeks of intratibial injection of 143B. In addition, other studies found that plasma EV-miR-101 is able to distinguish between OS patients with metastasis from those without. Insulin like growth factor (IGF)2, was reported to be shuttled by EVs and was isolated from biological fluids of healthy subjects as well as of cancer patients (62). Moreover, OS survival is sustained by IGF2, that induced dormancy by triggering autophagic pathways and inducing chemoresistance to adriamycin in syngenic animal models injected with the OS murine ATX cells (117). Notably, in biopsies from OS patients with favorable prognosis after chemotherapy, IGF2 levels are decreased.

**Table 2:** Extracellular vesicles in the support of tumor dormancy in the bone.

Donor	EV-Mediator	Target	Effect	Refs
EWS cells A673,SK-ES-1	miR-210	Autologous	CASP8AP2 silencing	(108)
SAOS2, SOSP-9607, 143-B	miR-101	Autologous	BCL6 silencing	(109)
AXT osteosarcoma cells	IGF2	Autologous	Autophagy induction	(110)
MSCs	miR-23b	Breast cancer cells BM2	*MARKS* suppression	(111)
Breast cancer cells MDA-MB-231, T47D	miR-222/223	MSCs/autologous	Switch in G1-G0 phase	(112)
Breast cancer cells MDA-MB-231, T47D	Whole EVs	M1/M2 macrophages	Cancer proliferation/dormancy	(113)
MSCs	miR-31/-205	Breast cancer cells MDA-MB-321	*UBE2N* suppression	(114)
MSCs	Whole EVs	Breast cancer cells MCF7	Dormancy induction	(115)
Endothelial cells	miR-126	Leukemic cells	Dormancy induction	(116)

With regards to bone metastases (**Figure 2** and **Table 2**), in an *in vitro* study Ono et al. found that EVs from MSCs decrease the proliferation, invasion, and sensitivity to chemotherapeutics of the bone metastatic human BrCa BM2 cells (63). These effects were due to the shuttling of miR-23b from MSC-EVs to cancer cells, which suppresses *MARKS* (myristoylated alanine-rich C kinase substrate) expression, encoding for a protein that promotes cell cycling and motility. Likewise, a study from Bliss demonstrated that BrCa cells promote the MSCs release of EVs enriched in miR-222/223, which in turn arrested into G1-G0 phase a subset of cancer cells, thus favoring a quiescent phenotype. In contrast, *naïve* MSC-derived EVs induced MDA-MB-231 cells into cycling (64). The same group dissected the crosstalk of the bone marrow macrophages with dormant BrCa cells, finding that M2 macrophages support the dormancy of BrCa cells through gap junctions, arresting tumor cell cycle. Interestingly, Walker et al. demonstrated that M1 macrophages release EVs able to awake cancer cells and induce proliferation, migration, and epithelial to mesenchymal transition (65). Another study revealed that MSCs support the dormancy of MDA-MB-321 through the EV-mediated shuttling of miR-31 and 205 (66). These miRNAs target *ubiquitin conjugating enzyme E2 N* (*UBE2N*) gene, suppressing the proliferation, migration, and invasion of tumor cells in bone. Similarly, proliferation and migration ability were reduced in MCF7 BrCa cells treated with MSC-EVs while enhancing cell adhesion (118).

## Extracellular Vesicles in Bone Cancer Drug Resistance

Overcoming chemoresistance is a major clinical unmet need, since what is usually the first line treatment is not an option for patient with chemoresistant cancer. Many mechanisms have been elucidated in the establishment of chemoresistance, and EVs have been shown to shuttle the necessary molecular machinery (**Figures 1**, **2** and **Table 3**) to promote drug resistance (131).

**Table 3:** Extracellular vesicles in cancer drug-resistance in the bone.

Donor	EV-Mediator	Target	Effect	Refs
MG-63	P-gp1	Autologous	Resistance to doxorubicin	(119)
Osteosarcoma biopsies, 143B, U2-OS	miR-25-3-p	Autologous	Dkk3 silencing	(120)
Osteosarcoma HMPOS cells,canine osteosarcoma biopsies	Tetranectin	Autologous	Resistance to cisplatin	(121)
Seric EVs from patients,MG63, U2OS cells	circRNA103801	Autologous	Resistance to cisplatin	
Breast cancer cells MCF7	P-gp1	Autologous	Resistance to docetaxel	(122)
Breast cancer cells MCF7	UCH-L1	Autologous	Resistance to doxorubicin	(123)
Breast cancers cells MCF7	miR-222	M2 macrophages	*PTEN* silencing and M2 proliferation	(124)
Breast cancer cells MCF7	miR-222	Autologous	Resistance to tamoxifen	(125)
Breast cancer cells MCF7	GSTP1	Autologous	Resistance to doxorubicin	(126)
MDA-MB-231	Survivin	Autologous	Resistance to paclitaxel	(127)
Breast cancer cells SKBR3 and BT474	HER2	Autologous	Resistance to trastuzumab	(128)
CAFs	5’-triphospate RNA	Breast cancer cells	Radiation- and chemo-resistance	(129)
CAFs	miR-423-5p	prostate cancer cells(LNCAP, 22RV-1, C4)	Resistance to taxane	(130)

### Drug Resistance in Bone Tumors: Role of EVs

Primary OS MG63 cells treated with doxorubicin (DXR) increased their expression of P-glycoprotein (P-gp)1, a membrane transporter pumping xenobiotics outside the cell. Consistently, EVs from DXR treated MG63 presented with higher levels of both *ABCB1* transcript and P-gp encoded pretein expression, that can be transferred to untreated MG63 cells, conferring them drug resistance (132). In another study, upregulation of miR-25-3p, which silences the Dickkopf WNT signaling pathway inhibitor 3 (DKK3) gene, was detected in human OS samples, where it is negatively correlated with clinical outcome (133). The same authors demonstrated that miR-25-3p upregulation supported tumor growth and drug resistance and that the same effects were observed after DKK3 silencing. Interestingly, miR-25-3p was found in cell-derived EVs. In another study by Weinman et al. the correlation between drug resistance and EVs has been investigated in a spontaneous canine model of OS (134). The authors clustered the animals in two cohorts based on the responsiveness to amputation and adjuvant carboplatin chemotherapy (good, disease-free interval > 300 days; poor, disease-free interval < 100 days) and analyzed the protein profile of circulating EVs by mass spectrometry. The proteomic profile revealed differences in EV cargo, identifying tetranectin, which was decreased in the poor prognosis group, as the most reliable biomarker.

The clinical relevance of EV-mediated drug resistance in OS patients has been investigated by Pan et al. (135). The analysis of circulating EVs from 43 OS patients compared to healthy subjects revealed overexpression of the circular RNA circRNA103801 in the former. The level of this circRNA presented a prognostic value, being inversely correlated with the overall survival of patients. The authors further investigated the biological function of the circRNA103801 in an *in vitro* model of OS. The overexpression of circRNA103801 in MG63 cells increased the sensitivity to cisplatin, the OS cells released also EV enriched in the circRNA. When these EVs were incubated to naïve MG63 and U2OS cells, both the OS cells increased the sensitivity to cisplatin, upregulating the expression of P-gp and Multidrug Resistance Protein 1, MRP1. These experiments demonstrated that circRNA103801 is responsible for conferring chemoresistance in OS patients.

### Drug Resistance in Bone Metastases: Role of EVs

Like in OS, the expression P-gp was increased in MCF7 BrCa cells after exposure to docetaxel (DOC) Moreover, EVs from DOC-treated MCF-7 expressed higher levels of P-gp compared to EVs from *naïve* MCF-7, and the incubation with DOC-MCF-7 EVs reduced cell apoptosis of *naïve* MCF-7 (108). It is known that *ABCB1* can be activated by the ubiquitin carboxy-terminal hydrolase (UCH) L1 through MAPK/ERK pathway. Consistently, MCF7 treated with adriamycin (ADR) show high level of UCH-L1 and phospho-ERK compared to control cells and when *naïve* MCF7 cells were cultured with EVs from ADR-MCF7 they acquired a reduced sensitivity to ADR and increased p-ERK and P-gp levels (109). Interestingly, EVs from blood of BrCa patients were positive for UCH-L1 and show an inverse correlation with chemosensitivity.

Chemotherapeutics resistance can also occur *via* EV-mediated miRNAs transfer. In fact, ADR/DOC-resistant BrCa cells release EVs enriched in miR-222, suppressing phosphatase and tensin homolog (*PTEN)* gene, a tumor suppressor which negatively regulates intracellular levels of phosphatidylinositol trisphosphate and the Akt signaling (136). These EVs are also taken up by M2 macrophages, eventually promoting their activation and polarization to support cancer cells. Accordingly, miR-222 has been also found in EVs from plasma and tissue of chemoresistant patients (110). Exosomal miR-222 is also responsible for the resistance to tamoxifen in MCF7 cells, suppressing p27 and Estrogen Receptor (ER) alpha expression (110).

Cancer cells can counteract chemotherapeutics like ADR by overexpressing the glutathione-S-transferase P1 (GSTP1), a phase II-metabolizing enzyme that detoxifies chemicals by conjugating with glutathione. Yang et al. found that this enzyme is present in EVs from ADR/MCF7 and sera of chemoresistant patients (111). Another study described that, when exposed to paclitaxel (PTX), MDA-MB-231 specifically released EVs enriched in Survivin, an inhibitor of apoptosis (112). The protective effects of Survivin-enriched EVs were effective on drug-sensitive fibroblasts and SKBR3 cells when exposed to PTX. Furthermore, tumor cells can directly counteract chemotherapeutics by EV release, *via* a decoy-like system. Indeed, Ciravolo et al. described that the human epidermal growth factor receptor 2 (HER2)-positive BrCa cells SKBR3 and BT474 release HER2-positive EVs. These EVs are able to bind and neutralize the biological drug trastuzumab, an anti-HER2 antibody, while EVs from triple negative BrCa cells MDA-MD-231 do not (113). In fact, SKBR3 cells treated with autologous EVs were less sensitive to the anti-proliferative effect of trastuzumab. Interestingly, the authors found that EVs purified from sera of HER2-positive BrCa patients showed lower binding to trastuzumab compared to EVs circulating in sera from patients with advanced disease.

Notably, cancer cells can also take advantage of CAFs derived EVs to acquire chemoresistance. Indeed, it has been shown that EVs from CAFs sustain radiation- and chemo-resistance of MDA-MB-231 BrCa cells by activating retinoic acid-inducible gene I (RIG-I), signal transducer and activator of transcription (STAT) 1, and NOTCH3 pathways (114). Similarly, Shan *et al.* dissected the role of CAF-EVs in taxane resistance-acquisition by PrCa cells, finding an enrichment of miR-423-5p in these EVs which, once internalized in PrCa cells (LNCAP, 22RV-1 and C4 cells) suppressed *GREM2* (encoding for gremlin2 protein inhibitor of bone morphogenetic proteins family members, BMPs) and increased TGF-β eventually leading to a reduced sensitivity to taxane (115).

## Potential Applications of EVs in Clinical Management

Cancer-derived EVs can be also used for diagnosis and to monitor cancer progression, exploiting them in the field of liquid biopsy. Of note, fibronectin shuttled by EVs isolated from BrCa patients is becoming a reliable diagnostic marker for detecting tumor early stages (116). On the same line, developmental endothelial locus-1 protein (Del-1) has been proposed as an exosomal biomarker to discriminate between benign and malignant BrCa (119). Periostin is another potential EV-related biomarker, since it has been found enriched in circulating exosomes from BrCa patients, and allowing the stratification of patients with localized disease *versus* those with lymph node metastasis (120). HER2 expression in circulating EVs has been found to be consistent with the positivity assessed by immunohistochemistry on tumor biopsies (121). Similarly, PrCa diagnosis can be addressed *via* EVs, since the classical marker prostate specific antigen (PSA) can be measured in EVs from patients, distinguishing benign hyperplasia from malignant transformation (137). EV-bound survivin has been proposed as a biomarker and clinical tool for diagnosing or monitoring PrCa as well as PTEN, both being detectable only in patients (138). Melanoma can be also monitored by circulating EVs profile of caveolin 1, and lung cancers can be histologically discriminated through a multimarker model based on the expression on circulating EVS of tetraspanin 8/CD151/CD171 (122, 139). Not only protein cargoes but also miRNA profiles of circulating EVs in patients suffering from cancers are pursued for prognostic and diagnostic aims (123–126).

Another useful approach in the EVs field is their employment as pharmacological delivery system for the treatment of different cancers (127). Indeed, EVs present with attractive potentialities due to a relatively long half-life, high biocompatibility, and minimal or no adverse effects. As an example, Melzer et al. showed the efficacy of taxol-loaded MSC-derived exosomes in targeting MDA-MB-231 growth and metastases in NOD-SCID mice (128). Similarly, zoledronate and dasatinib were efficaciously delivered by osteoblast-EVs and suppressed osteoclast formation and function *in vivo*, thus potentially being efficacious in reducing tumor-induced osteolysis (31). Consistently, EVs from the monocyte/macrophage cell line RAW264.7 have been reported to be efficient vehicles for paclitaxel and doxorubicin shuttling and treatment of mice orthotopically injected with MDA-MB-231 cells (129).

Finally, EVs are promising tools for immunotherapy (130). As an example, EVs derived from overexpressing IL-12 transgenic renal and bladder cancer cells efficiently trigger a strong antitumoral activity of cytotoxic T cells through the FasL/Fas signaling pathway, and the same strategy could be applied to bone primary tumors (140). Xu’s group demonstrated that EVs from macrophages carry phagocytosed antigens to dendritic cells and strengthen T-cell responses, conferring anti-tumor immunity (141). Dendritic cell-derived EVs induced specific cytotoxic T cell activation suppressing the growth of lung adenocarcinoma in a xenograft mouse model (142), while Zhu et al. found that NK cell primed by IL-15 shuttled-EVs show a higher cytolytic effect against BrCa cells MDA-MB-231 injected in nude mice (143). Many other studies and ongoing clinical trials are endorsing these approaches.

## Outlook and Perspectives

The whole body of evidence from the literature strongly indicates that EVs exert a critical role in the progression of both primary and secondary bone tumors. Bone is an attractive microenvironment for tumor cells growth and metastasis, through the establishment of a crosstalk among many different resident cell types, occurring *via* juxtacrine, paracrine, and EV-mediated mechanisms. The latter are an additional piece of the complex system by which tumor fuels this “vicious cycle”. The translational significance of this aspect is endorsed by the growing interest of the application of EVs in diagnostic and therapeutic fields, as well as the patent applications/grants and clinical trials based on the use of EVs. However, many challenges are still present in the field. For the basic research side the main issues are: i) to isolate with very high purity the different EV subpopulations, ii) to identify specific mechanisms of selective EV uptake into target cells, iii) to track the kinetics and distribution of EVs towards distant tissues, and iv) to distinguish the real contribution of the molecular legacy of EVs into an complex microenvironment, such as the tumor *milieu*. The isolation and quantification methods are still a crucial issue. Depending of the technique used for the isolation, the composition and the effect of the EVs from the same source can be different, and the same goes for the quantification. Of course, there are consensus methods in the field, but every method presents pitfalls to take into account. Protein titration, one of the most widely used methods in laboratory practice, can be affected by “exogenous” contaminants and does not consider the size distribution. Methods of quantification based on physical features, such as dynamic light scattering and resistive pulse sensing, cannot estimate the entire EV population, since generally a size cut-off must be applied on the instruments, and cannot distinguish between intact and damaged EVs.

The possible experimental approaches to dissect biological aspects of EVs have pros and cons: the *in vitro* studies can help to quantify and estimate the contribution of a specific pathway in the cellular context. However, the *in vitro* cell system cannot take in consideration the paracrine and systemic contribution of all cell types on cancer metabolism and related EVs. Furthermore, the EV cargo is a balanced cocktail of molecules that can exert opposite effects (i.e., EVs can shuttle both RANKL and OPG), and generally the investigators focus only on one member of them, possibly overestimating the importance of the single factor using targeted rather than system/pathway-oriented overexpression or knock-down techniques. On the other side, the *in vivo* studies in animal models can help to assess the real contribution of a molecular player on a biological system, but many confounding factors are present due to the complexity of the system. The *in vivo* studies help to investigate the kinetics of release and integration of EVs, frequently using intravital microscopy approaches. These studies provide impactful data, but are limited by technical constraints, such as resolution, signal/noise ratio and cellular recycling of the tags used for tracking the EVs.

Finally, a real clinical setting of EVs is hindered by many limitations. The main technical issues are shared with basic research. Indeed, a lack of standardization methods for isolation, characterization, quality control, large scale production and storage conditions reduce the translational value of EVs (144). Moreover, the EVs and their molecular profile can change for several patient-related variables, including the circadian rhythm and lifestyle habits as well as comorbidities, affecting their prognostic reliability (145, 146).

In conclusion, EVs are an active field of study for a better understanding of the biological bases of tumorigenesis, but also offer a promising translational tool for diagnosis, monitoring, and treatment of cancer patients.

## Author Contributions

AC and NR contributed to original draft conceptualization, preparation, and revision. Both authors have read and agreed to the final version of the manuscript. All authors contributed to the article and approved the submitted version.

## Funding

“Associazione Italiana per la Ricerca sul Cancro” (AIRC, #IG 2020 ID 24823) to NR.

## Conflict of Interest

The authors declare that the research was conducted in the absence of any commercial or financial relationships that could be construed as a potential conflict of interest.

## Publisher’s Note

All claims expressed in this article are solely those of the authors and do not necessarily represent those of their affiliated organizations, or those of the publisher, the editors and the reviewers. Any product that may be evaluated in this article, or claim that may be made by its manufacturer, is not guaranteed or endorsed by the publisher.
